# Scrub Typhus: No Longer Restricted to the Tsutsugamushi Triangle

**DOI:** 10.3390/tropicalmed3010011

**Published:** 2018-01-25

**Authors:** Ju Jiang, Allen L. Richards

**Affiliations:** Viral and Rickettsial Diseases Department, Naval Medical Research Center, 503 Robert Grant Avenue, Silver Spring, MD 20910, USA; Ju.Jiang2.ctr@mail.mil

**Keywords:** scrub typhus, *Orientia tsutsugamushi*, Tsutsugamushi Triangle, endemic region

## Abstract

Scrub typhus is the most important rickettsial disease in the world. Its previous endemic region was considered to be in Asia, Australia and islands in the Indian and Pacific Oceans; this area was referred to as the Tsutsugamushi Triangle. Accumulation of serological, molecular, genetic, and culture data have shown that not only is scrub typhus not limited to the Tsutsugamushi Triangle, but can be caused by orientiae other than *Orientia tsutsugamushi*. This review describes evidence currently available that will be instrumental to researchers, healthcare providers and medical leaders in developing new research projects, performing diagnosis, and preventing scrub typhus in locations not previously thought to be endemic.

## 1. Introduction

Scrub typhus is today’s most important rickettsial disease, worldwide. Approximately one million cases occur each year, and over one billion people are at risk of disease [1]. Scrub typhus is a mild to life-threatening disease with a fatality rate without treatment as high as 50%. Disease presentation consists of abrupt high fever, severe headache, lymphadenopathy, generalized myalgia, eschar, and rash. The eschar, a painless lesion at the site of the bite of an *Orientia tsutsugamushi*-infected *Leptotrombidium* chigger mite is considered pathognomonic; it is seen a few days after the chigger bite, but before disease presentation, and is therefore an important early sign associated with scrub typhus. Though many professional phagocytic cells (dendritic cells, macrophages, neutrophils) may be infected by *O. tsutsugamushi*, the ultimate target cells are the endothelial cells, so all tissues and organs of the body can be infected; thus the presentation of various manifestations of scrub typhus, including pneumonitis, mild hepatitis, tinnitus, rash, disseminated intravascular coagulation, and meningoencephalitis, can occur [2]. The previous geographic distribution of scrub typhus included many areas of China, Japan, Indonesia, Malaysia, Thailand, Pakistan, Korea, northern Australia and the islands of the western Pacific and Indian Oceans [3,4,5,6]. This area has been described as the Tsutsugamushi Triangle [3,4,5,6,7].

## 2. Scrub Typhus outside of the Tsutsugamushi Triangle

### 2.1. Serological Evidence of Orientia Species Infection in Africa

Evidence has been slowly accumulating for decades that scrub typhus may not be restricted to just the Tsutsugamushi Triangle. As early as 1951, Giroud and Jadin [8] presented evidence of scrub typhus outside the Tsutsugamushi Triangle in eastern Africa. They noted that native Africans from Runda-Urundi (formerly the Belgian Congo and now Rwanda and Burundi), who presented with fever in Musha Hill, had skin hypersensitivity to rickettsial antigens, including two individuals who reacted to *Rickettsia orientalis* (*O. tsutsugamushi*) antigens. To confirm the skin tests, blood from the two patients was tested for complement-fixing antibodies to *O. tsutsugamushi*, and was found to be positive with titers of 80 and 320. To assess the reactivity to the scrub typhus assays in other populations who lived close to those native Africans in Musha Hill, healthy individuals, including nine people born in Muscat, Oman, five born in Bombay, India, and two born in Africa whose parents were born in Bombay, were tested using the same skin and blood tests for evidence of previous *O. tsutsugamushi* infection. Muscat and Bombay were considered by the authors to be scrub typhus-endemic regions in which people might be antibody positive to *O. tsutsugamushi*, and therefore act as positive controls. Of the nine individuals originally from Muscat, three individuals each had strong, weak, and negative skin reactivity; and from eight of the nine individuals, antibodies against *O. tsutsugamushi* were detected with titers of 1280 (four individuals) and 640 (three individuals). From the five people born in Bombay, three were skin reactive positive to *O. tsutsugamushi* antigens; and interestingly, the two people whose parents were from Bombay, but who were born in and never traveled outside of eastern Africa, were also positive. These results suggested the presence of scrub typhus in eastern Africa. The authors indicated that a similar study they conducted among natives in western Africa was negative for evidence of scrub typhus [8].

In the 1990s, three case reports also suggested the presence of scrub typhus in Africa. The first described an individual from Japan visiting the Republic of Congo, who presented with fever six days after his return from Africa [9]. The disease was identified as scrub typhus, though the possibility could not be ruled out that the patient had contracted scrub typhus in Japan, an endemic country for scrub typhus, where the patient resided during those six days. The second case was a US missionary who visited Cameroon [10]. Within two weeks of his visit the missionary noted a lesion on his leg and three days later he had fever and noted a rash. Two weeks later, the missionary returned to the US and was subsequently admitted to a hospital in which he was treated for a rickettsial disease. He recovered within 24 h with doxycycline treatment, and he had a four-fold increase in titer of antibodies against *O. tsutsugamushi* between his acute and convalescent serum samples, from 256 to 1024, respectively. The third case was that of an individual who had visited Tanzania [11]. She had noted a lesion on her right foot, and had a three-day history of fever and headache after returning to the Netherlands. Her acute and convalescent sera showed antibody seroconversion to *O. tsutsugamushi* antigens from <16 to 1024 by IFA. Unfortunately, none of these three cases had produced a culture of orientia or molecular evidence of the causative agent(s).

More recently, additional serological evidence for the presence of orientia infections in eastern Africa has been presented in three articles. In the first of two studies conducted in Kenya, single serum samples from individuals presenting with fever at various hospitals throughout the country were assessed for antibodies against *O. tsutsugamushi* ELISA antigens. Seroreactivity was found in 70 of 1401 (5%) patients, and was confirmed by Western blot assays [12]. The second report was of a fever study conducted among febrile children (1–12 years of age) admitted to Webuye District hospital in western Kenya, from whom paired acute and convalescent serum samples were assessed for causes of their illnesses. Fifteen of 281 patients (5.8%) had antibodies against *O. tsutsugamushi* ELISA antigens, and 10 of these children seroconverted (3.6%). The seroreactivity was confirmed by Western blot analysis [13]. The third report involved a 20-week investigation of arthropod-borne and zoonotic diseases among abattoir workers in Djibouti [14]. Three of 49 workers had antibodies against *O. tsutsugamushi* ELISA antigens, and one individual reported a history of a febrile disease and seroconverted to orientia antigens by ELISA, IFA and Western blot tests. Collectively, these serological reports added considerably to the growing evidence of the presence of scrub typhus in Africa.

### 2.2. Molecular Evidence of Orientia Species in Africa and Europe

In 2015, the first molecular evidence of orientiae in Africa was provided by Cosson et al [15]. The authors showed fragments (251 bp) of the 16S rRNA gene sequence targeting the hypervariable region 4 by the Illumina MiSeq system were similar to *Orientia* sequences from GenBank. The gene sequences were detected in spleen tissues of 48 of 207 (23%) house mice (*Mus musculus*), but not from any of 147 rats (*Mastomys erythroleucus*) captured from a region along the Senegal River, Senegal. The authors also reported that 52 of 415 (12.5%) rats (*Arvicola sherman, Myodes glareolus*, and *Microtus arvalis*) collected from the Ardennes region, France, were positive for 16S rRNA gene sequences similar to orientiae.

**Figure 1:**
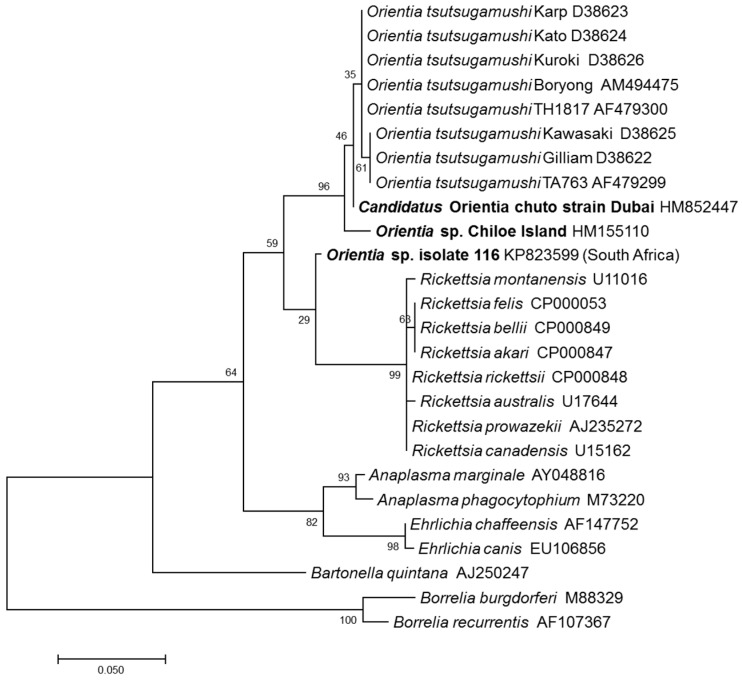
Evolutionary relationships of *Orientia* species (in bold) detected outside of the Tsutsugamushi Triangle compared with *Orientia tsutsugamushi* strains and *Rickettsia* species (GenBank accession numbers are shown next to each agent). The tree was based on 256 bp *rrs* gene fragments and constructed using the Maximum Likelihood method based on the Tamura-Nei model. Evolutionary analyses were conducted in MEGA7 and the values for the bootstrap test (1000 replicates) are shown next to the branches. The tree is drawn to scale, with branch lengths measured by the number of substitutions per site.

A second report of molecular evidence of orientiae in Africa came from a healthy dog from Mnisi, Bushbuckridge, Mpumalanga Province, South Africa [16]. The dog’s blood sample had 16S rRNA sequence that was 96.1% (247/257 bp) similar to that of *Orientia*. This sequence was placed between *Orientia* and *Rickettsia* based upon phylogenetic relationship analysis (Figure 1).

### 2.3. Scrub Typhus Cases in the Middle East and South America

The first definitive case of scrub typhus that occurred outside of the Tsutsugamushi Triangle, was reported in 2010 [17], in which a patient who had visited Dubai, United Arab Emirates, reported a lesion on her abdomen and subsequently fever, prior to returning to Australia. She was admitted and investigated for rickettsial diseases. Her acute blood sample prior to treatment was sent to the laboratory for culture and tested for antibodies against *O. tsutsugamushi*. The blood sample grew an orientia that was characterized genetically by sequencing gene fragments of the 16S rRNA gene (*rrs*), the 47 kDa HtrA gene (*htrA*), and the 56 kDa TSA gene (*tsa*). The sequences for *rrs*, *htrA* and *tsa* were only 98.5%, 82.3% and 53.1% similar to the closest sequences of orientiae in GenBank. Thus, this agent was considered to represent a new species of *Orientia*, that is, *Candidatus* Orientia chuto. The patient’s paired serum sample showed a four-fold increase in antibody titer from 512 to 8192, confirming the patient had scrub typhus. It is interesting to note that Dubai is less than 500 km away from Muscat, Oman, where the individuals came from who tested positive for antibodies against *O. tsutsugamushi* antigens in the Giroud and Jadin report [8]. Also of note was the fact that, following treatment with doxycycline, the patient remained febrile for up to 10 days. This may have been due to delay in treatment, confounding medical factors, virulence of the agent, and/or antibiotic resistance that maybe unique to this agent or area of the world [2,3].

A second report of a scrub typhus case outside the Tsutsugamushi Triangle was that of a biologist working in Chiloè Island in southern Chile [18]. The individual reported being bitten by leeches, but not by ticks or mites. The patient had an eschar and febrile disease that responded to doxycycline treatment. Additionally, the acute and convalescent sample showed a seroconversion by IgG antibody against *O. tsutsugamushi* ELISA antigens (titer <100 to 400). Moreover, the partial gene sequence of *rrs* obtained from the patient’s eschar and rash biopsy was only 96.5% similar to the closest agent in GenBank, *O. tsutsugamushi*, whereas the *rrs* sequence divergence among *O. tsutsugamushi* strains is between 0.1 and 1%. Phylogenetic analysis based on the *rrs* showed *Ca*.*O. chuto* and the *Orientia* sp. from Chiloè Island were separate from *O. tsutsugamushi* species, but clearly closer to the strains classified within *O. tsutsugamushi*, compared with other rickettsiae (Figure 1).

Last year, a report of three more cases of scrub typhus that occurred in 2015 and 2016 among individuals residing in Chiloè Island was presented [19]. Again, the cases offered signs and symptoms of scrub typhus, including eschars and rash, that were observed in all three individuals (Table 1), and two of the patients had paired serum samples that either showed a seroconversion or a four-fold rise in antibody titer. Only a single convalescent sample from the third individual was available and was tested. It showed a high antibody titer against *O. tsutsugamushi* antigens. In addition, two cases were PCR positive for scrub typhus, and one patient had a 56 kDa *tsa* sequence similar to *O. tsutsugamushi*. Additional evidence for the presence of scrub typhus in South America was recently described from Peru [20]. The authors reported that, of 1,124 individuals enrolled in a febrile surveillance study in Iquitos, Peru, near the Amazon River, 60 (5.3%) were seropositive against *O. tsutsugamushi* ELISA antigens, and one person had a four-fold increase in titer, suggesting that he had scrub typhus. The ELISA results of this sample were confirmed by IFA.

## 3. Conclusions

**Figure 2:**
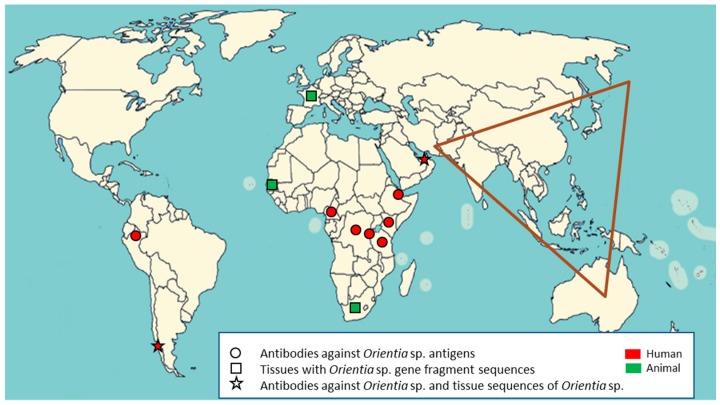
Human and animal serological and molecular evidence of *Orientia* spp. infections in new endemic regions of scrub typhus outside of the Tsutsugamushi Triangle (shown in brown).

Collectively, all of these reports of scrub typhus evidence from Africa, France, the Middle East, and South America, as shown in Figure 2, lead to the supposition that we should no longer consider the Tsutsugamushi Triangle to be the only endemic region for scrub typhus. Moreover, we should acknowledge that there are new *Orientia* species, and potentially new vectors for scrub typhus, that have yet to be discovered.

## Figures and Tables

**Table 1:** Scrub typhus patient presentations outside of the Tsutsugamushi Triangle.

Patient	Country of Origin	Area/Country Acquired Diseases	Signs and Symptoms	Laboratory Results	Treatment/Time to Defervescence	Reference
46-year-old male	United States	Rural village/Cameroon, West Africa	Eschar on left lower leg, fever (39.6 °C), rash initially on legs, headaches, myalgias, and malaise	Decreased platelet count and WBC (leukopenia)	Doxycycline/24 h	[8]
44-year-old female	Netherlands	Rural areas/Tanzania, East Africa	Eschar on right foot, fever (39.8 °C) and headache	Elevated erythrocyte sedimentation rate	Ciprofloxacin 1 week after noticing the eschar, no effect; doxycycline/not reported	[9]
52-year-old female	Australia	Dubai (stable)/United Arab Emirates (UAE), West Asia	Eschar on abdomen, fever and rash, lymphadenopathy, myalgia, headache, pain behind eyes, backache	Elevated WBC and C-reactive proteins (CRP); abnormal liver function: elevated alanine aminotransferase (ALT), aspartate aminotransferase (AST) and alkaline phosphatase (ALP), and elevated gamma-glutamyl transpeptidase (GGT)	Doxycycline/10 days	[15]
54-year-old male	Chile	Chiloé Island/Chile, South America	Eschar on left leg, high-grade fever (39.0 °C), headache, myalgia, and scanty dry cough, rash; bilateral conjunctival suffusion	Abnormal liver function: elevated ALT and AST	Doxycycline/3 days	[16]
38-year-old female	Chile	Chiloé Island/Chile, South America	Eschar on abdomen, maculopapular rash, fever (40.0 °C), headaches, and intense myalgia especially in the calves, malaise and confusion, apathetic, bilateral conjunctivitis	Elevated erythrocyte sedimentation rate, abnormal liver function: elevated CRP, ALT, AST and GGT	Doxycycline/24 h	[17]
40-year-old male	Chile	Chiloé Island/Chile, South America	Eschar on right leg, rash, high fever, chills, night sweats, headaches, myalgia, retro-orbital pain and photophobia	Abnormal liver function: elevated CRP, ALT and AST	Cloxacillin and anti-inflammatory, no effect; doxycycline/24 h	[17]
55-year-old male	Chile	Chiloé Island/Chile, South America	Eschar on upper left thigh, rash, high fever, chills, night sweats, intense headaches, myalgia, and arthralgia	Elevated CRP	Fever and systemic symptoms resolved spontaneously after 1 week; doxycycline/not reported	[17]

## Disclaimers

The views expressed in this presentation are those of the author, and do not necessarily reflect the official policy or position of the Department of Navy, Department of Defense, nor the U.S. government. A.L.R. is an employee of the U.S. Government. This work was prepared as a part of his official duties. Title 17 U.S.C. §105 provides that ‘Copyright protection under this title is not available for any work of the United States Government’. Title 17 U.S.C. §101 defines a U.S. Government work as a work prepared by a military service member or employee of the U.S. Government as part of that person’s official duties.
